# Pushing the Fœtus through the Pelvis

**Published:** 1871-02

**Authors:** S. B. Hawley

**Affiliations:** Aurora, Illinois


### PUSHING THE FŒTUS THROUGH THE PELVIS.

Read before the Fox River Medical Society, January 9, 1871, by S. B. HAWLEY, M.D., Aurora, Illinois.

For fifteen years I have been in the practice of applying pressure with the extended hand over the umbilical region during the pains of labor, in those cases where a contracted pelvis or a large head delayed labor, after a full dilitation of the os uteri had taken place. And, I have many times felt fully satisfied, that I had seen highly beneficial results, both to mother and child, in saving time and suffering, as well as avoiding the the necessity of instrumental interference in many cases that would otherwise require it.

But recently a case has occurred in my practice, the history of which, in connection with the patient's former experience, so clearly demonstrates the wisdom and mercy of the practice, that I feel it my dnty to report it.

Mrs. S., in her first labor, was attended by two of the best physicians in our city, who, after a very protracted waiting for nature to deliver the patient, when exhaustion was manifest, delivered her with the forceps, losing the child, and hardly saving the life of the mother.

Her second gestation was terminated by a similar result, though in the hands of a skilful accoucher.

I first learned these facts at her bedside, after the commencement of her third labor. I found the rational cause of these difficulties to be a contraction of the superior strait of the pelvis, in its antero posterior diameter, about three-fourths of an inch below the average; so that, upon my second examination, although the os was so well dilated as to interpose no obstacle to the advance of the head, it still remained above the upper strait, with seeming small promise of entering it at all.

I at once resolved not to wait for nature to exhaust herself, but to supplement her efforts while they were the strongest, and, accordingly, placing my patient on her right side, near the edge of the couch, I took my station behind her, and, with each returning pain, I applied my right hand, with fingers extended to cover as much surface as possible, to the fundus uteri, and pressed, with all the force I could exert, in a line at right angles with the plane at the upper strait of the pelvis. It was not long till signs of progress were manifest, and in three hours time she was delivered of a living child, which weighed eight and a half pounds. Though the labor had lasted but this short time, the head was much elongated and compressed, showing that it had been forced to yield very much by the force employed. I have no doubt that had I waited upon nature's efforts, I should have been forced to the use of the forceps, at least, if not to a more objectionable resort.

I gave the patient chloroform, aiming to maintain the point where she would respond when spoken to, though not always correctly. In which state, I have frequently felt that all the muscular powers of the patient were more fully exerted to accomplish the delivery, than they could be when a sense of the suffering produced by the efforts would produce a voluntary withholding of the bearing down effort.

In some cases I find this external pressure borne without complaint of pain, while, in some, it will not be endured unless under much suffering. In the latter class of cases, chloroform will be found of great advantage.

I have studied, with considerable care, the possibilities of good and bad results from this mode of aiding the parturient patient, and cannot discover any objection that does not hold with superior force against any mode of relief that can be adopted if nature fails in her efforts.

It has been estimated, that the direct expelling force exerted upon the fœtus, may be placed at a maximum of about 80 lbs., now, it is easy to supplement this, in the mode I have indicated, by at least 50 per cent., which, applied at each recurring effort of nature, must, by so much, increase the chance of success, by a process the nearest possible in imitation of nature's own plan. And, while the fœtus is closely enveloped in the contracting womb, it seems to me, that there must be far less danger to it, by force so applied, than there is in the use of forceps. It may be abused in unskilful hands, it is true, but it is useless for science to expect to so adapt her means, that they shall always work well, in whatever hands they may chance to fall, she, as well as nature, must demand conformity to her laws.

And, since so many women are found that have not that muscular strength, with which the hard-working wife of the peasant makes so easy a task of her labors in general, what reason is there why the accoucher should not, by his own voluntary effort, add the few pounds of pressure, which a better developed muscular system in the mother would so surely add, if occasion required. Of course, this should be done only when and where obstacles or delay seem imperatively to demand it.

